# Chemical Composition and Biological Activities of *Hedychium coccineum* Buch.-Ham. ex Sm. Essential Oils from Kumaun Hills of Uttarakhand

**DOI:** 10.3390/molecules27154833

**Published:** 2022-07-28

**Authors:** Sushila Arya, Ravendra Kumar, Om Prakash, Satya Kumar, Sonu Kumar Mahawer, Shivangi Chamoli, Piyush Kumar, Ravi Mohan Srivastava, Mozaniel Santana de Oliveira

**Affiliations:** 1 Department of Chemistry, College of Basic Science and Humanities, Govind Ballabh Pant University of Agriculture and Technology, Pantnagar 263145, Udham Singh Nagar, Uttarakhand, India; sushilaarya.sa2626@gmail.com (S.A.); oporgchem@gmail.com (O.P.); sonummahawer@gmail.com (S.K.M.); 2 Department of Plant Pathology, College of Agriculture, Govind Ballabh Pant University of Agriculture and Technology, Pantnagar 263145, Udham Singh Nagar, Uttarakhand, India; skumar8326@rediffmail.com; 3 Department of Biomedical Sciences, Vocational Studies and Skill Development, Central University of Haryana, Jant-Pali 123031, Mahendergarh, Haryana, India; shivangi.chamoli@gmail.com; 4 School of Health Sciences and Technology, University of Petroleum and Energy Studies, Bidholi 248007, Dehradun, Uttarakhand, India; piyush.kumar@ddn.upes.ac; 5 Department of Entomology, College of Agriculture, Govind Ballabh Pant University of Agriculture and Technology, Pantnagar 263145, Udham Singh Nagar, Uttarakhand, India; ravimohanento@gmail.com; 6 Campus de Pesquisa-Museu Paraense Emilio Goeldi-Botany Coordination, Av. Perimetral, 19001-Terra Firme, 66077-830 Belem-PA, Brazil

**Keywords:** natural products, bioactive compounds, (*E*)-nerolidol, 7-hydroxyfarnesen, biological activities

## Abstract

*Hedychium coccineum* Buch. Ham. ex Sm. is a perennial rhizomatous herb belonging to the family Zingiberaceae. The aim of the present study was to compare the chemical composition and biological activities of *H. coccineum* rhizome essential oil (HCCRO) and *H. coccineum* aerial part essential oil (HCCAO). The plant material was subjected to hydro-distillation using Clevenger’s apparatus in order to obtain volatile oil and analyzed for its chemical constituents using GC-MS. The comparative study of the rhizome and aerial part essential oils of *H. coccineum* displayed that (*E*)-nerolidol (15.9%), bornyl acetate (13.95%), davanone B (10.9%), spathulenol (8.9%), and 1, 8-cineol (8.5%) contributed majorly to the HCCRO, while 7-hydroxyfarnesen (15.5%), α-farnesene (11.1%), α-pinene (10.9%), spathulenol (7.7%), and β-pinene (6.8%) were present as major constituents in the HCCAO. Both the essential oils were studied for their biological activities, such as nematicidal, insecticidal, herbicidal, antifungal, and antibacterial activities. The essential oils exhibited significant nematicidal activity against *Meloidogyne incognita*, insecticidal activity against *Spodoptera litura*, and moderate herbicidal activity against *R. raphanistrum* sub sp. *sativus*, and good antifungal activity against *Fusarium oxysporum* and *Curvularialunata*. Essential oils were also tested for antibacterial activity against *Staphylococcus aureus* and *Salmonella enterica* serotype Typhi. Both oils showed good to moderate activity against the tested pathogens. The significant nematicidal, insecticidal, herbicidal, antifungal, and antibacterial activities of both the essential oils might be helpful for the development of environmentally friendly pesticides that could be an alternative to synthetic pesticides in the future.

## 1. Introduction

Zingiberaceae is an important plant family which has 52 genera and 1500 species, including *Hedychium coccineum* Buch.-Ham. ex Sm. Plants in the Zingiberaceae family are now being investigated extensively for their phytochemistry and pharmacological properties [1]. The genus *Hedychium* grows as an herb of perennial tuberous rootstocks, with aromatic flowers widely distributed in tropical and subtropical countries [2]. The species *Hedychium coccineum* Buch.-Ham. ex Sm. is a tall, herbaceous, and perennial herb commonly known as the scarlet ginger lily, scarlet ginger lily, orange ginger lily, and orange bottlebrush ginger, growing at the edge of forests and in mountain grasslands [3]. *H. coccineum* is intrinsic to the Himalayas of India and Nepal, China, Bangladesh, Myanmar, and Thailand [4]. The rhizome of the *H. coccineum* is used in the treatment of fever, headache, and body pain, and its flowers’ pulp is used on swollen body parts [5,6]. Indian tribal people believe that wearing the flower behind the ear could be effective against the evil eye and disease [7]. *H. coccineum* consists of a variety of active compounds and serves as a basis in science for the use of herbs as flavor and fragrance agents, food preservatives, botanical pesticides, nutraceuticals, and pharmaceuticals [8,9,10]. Phytochemical characterization of *H. coccineum* has been demonstrated to have an important role in recognizing its active principles, and has revealed that it is an important medicinal herb [11,12]. This herb is a source of valuable strong herbal medication and cures for various disorders due to the presence of various bioactive components [13]. *H. coccineum* rhizome essential oil composition and its biological activities seems to support further studies to describe it as a biopesticides or to develop new chemical compounds and the discovery of new pesticides. Thus, the present research aims to compare the chemical composition and biological activities of essential oils extracted from the rhizome and aerial parts of *H. coccineum.* This is the first report on chemical analysis and various biological activities of the *H. coccineum* essential oils from the Indian Himalayan region.

## 2. Results

### 2.1. Chemical Compositions of Essential Oils

**Figure 1:**
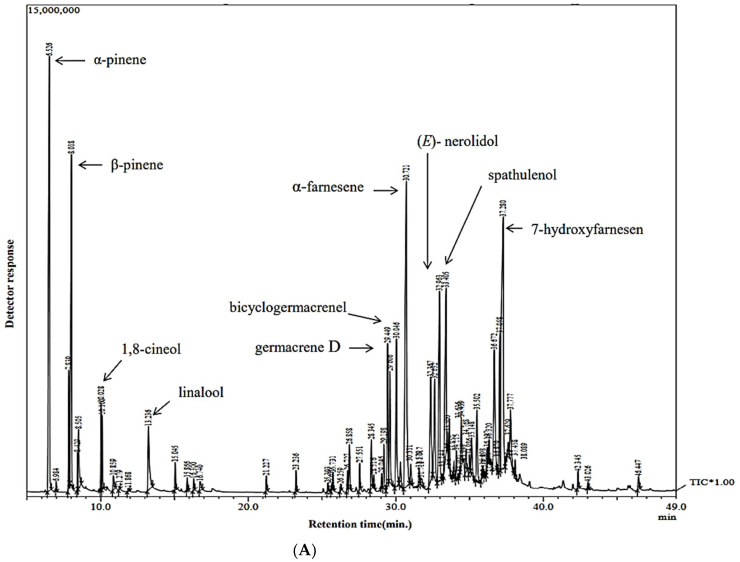

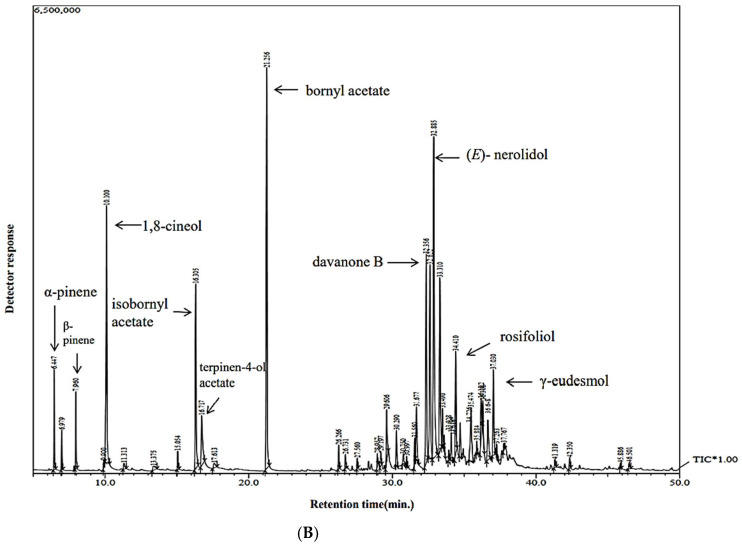
(**A**) Ion-chromatogram of HCCAO. (**B**) Ion-chromatogram of HCCRO. Relating to the chemical composition of essential oil fractions.

The phytoconstituents present in the HCCAO and HCCRO essential oils were identified, and are presented in Table 1 according to their order of elution on the DB-5 column in GC-MS. This is the first report to present a chemical analysis of the essential oil of *Hedychium coccineum* from the Indian Himalayan region. Fifty and thirty-two components were identified in HCCAO and HCCRO, contributing to 96.2% and 98.2% of the total volatile oils, respectively. 7-Hydroxyfarnesen (15.5%) and (*E*)-nerolidol (15.9%) were found to be a major constituent of HCCAO and HCCRO, respectively. Compounds that constituted more than 3.00% were considered main components, whereas compounds that constituted less than 3.00% were considered minor constituents in the essential oils under investigation. The ion-chromatogram of HCCAO and HCCRO essential oil is shown in Figure 1.

**Figure 2:**
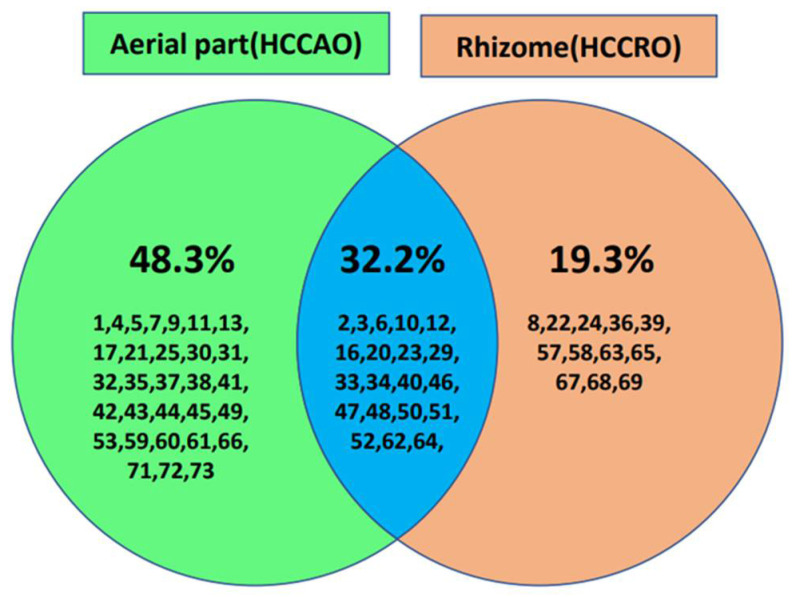
Venn diagram of essential oil composition of HCCAO and HCCRO (the chemical compositions were represented by different colors: HCCAO in green, HCCRO in orange, and common composition of both in blue).

In this study, it was observed that HCCAO was dominated by oxygenated sesquiterpene (39.1%), followed by sesquiterpene hydrocarbon (25.0%), monoterpene hydrocarbon (22.8%), and oxygenated monoterpene (9.3%), while HCCRO was dominated by oxygenated sesquiterpene (51.3%), followed by oxygenated monoterpene (32.5%), monoterpene hydrocarbon (9.5%), and sesquiterpene hydrocarbon (4.9%). Moreover, a Venn diagram (Figure 2) was generated to compare the chemical composition of HCCAO and HCCRO. It is exciting to observe that the essential oil of HCCRO has a remarkably different chemical makeup than HCCAO. Chemical constituents such as α-pinene, camphene, β-pinene, γ-terpinene, 1,8-cineol, linalool, camphor, terpinen-4-ol, bornyl acetate, α-*cis*-bergamotene, (*E*)-caryophyllene, α-curcumene, δ-cadinene, β-dihydroagarofuran, kessane, (*E*)-nerolidol, davanone B, spathulenol, guaiol, and τ-muurolol were present in both samples (HCCRO and HCCAO). However, *p*-cymene, *cis*-linalool oxide, *trans*-linalool oxide, *trans*-pinocarveol, *trans*-verbenol, borneol, α-terpineol, α-himachalene, β-acoradiene, globulol, viridiflorol, γ-eudesmolagarospirol, β-eudesmol, α-cadinol, and bulnesol were found to be unique to HCCRO, whereas sabinene, myrcene, artemisia alcohol, sulcatone, *cis*-sabinene hydrate, thujol, isoborneol, β-bourbonene, β-cubebene, β-elemene, γ-elemene, α–humulene, 9-epi-(*E*)-caryophyllene, α-neocallitropsene, germacrene D, bicyclogermacrene, α-farnesene, naphthalene, β-vetispirene, elemol, 7-hydroxyfarnesen, salvial-4(14)-en-1-one, rosifoliol, ledol, cadin-4-en-10-ol, α-(*Z*)-bergamotol, iso-longifolol, (*E*)-isovalencenol, 2-pentanone, and dodeca-(*2E,4E*)-dienal were present only in HCCAO, in variable amounts. These variations in the chemical compositions of essential oils obtained from different plant parts were supposed because of the direct contact with sunlight with HCCAO (aerial part), nonavailability of sunlight to HCCRO (rhizome), and different type of physiology in both type of plant parts. In other works, authors have reported that the chemical composition of essential oils can be affected by the presence of light, time of year, rain, dry weather, and circadian rhythm, among other factors [16,17,18]. The GC-MS profile and the detailed comparative chemical composition of HCCAO and HCCRO are represented in Table 1.

Table 1 represents the detailed comparative chemical composition of HCCAO and HCCRO in previously reported work [9,14]. The essential oil of *H. coccineum* has previously been reported in Mississippi, (U.S.) [9] and in Pamplemousses, (Mauritius) [14]. Sakhanokho et al. [9] revealed the presence of total 38 components contributing to 84.3% in *H. coccineum* rhizome part essential oil. The major constituents, such as linalool (26.7%), α-pinene (13.5%), bornyl acetate (8.4%), β-pinene (7.5%), (*E*)-nerolidol (4.6%), and α-curcumene (4.1%)were identified in the *H. coccineum* essential oil, whereas Gurib-Fakim et al. [14] revealed the presence of total 12 components, contributing to 77.6% in *H. coccineum* rhizome part essential oil. (*E*)-nerolidol (44.4%), *trans*-sesquisabinene hydrate(24.2%), and α-pinene (2.4%) contributed majorly to the composition of the *H. coccineum* essential oil sample [9,14].

Comparison of the results of present and previous investigations of *H. coccineum* rhizome essential oil revealed differences in their oil composition. A total of 50 and 32 components were identified to contribute to the composition of essential oils in the present study for HCCAO (96.2%) and HCCRO (98.2%), respectively. Meanwhile, only 38 and 12 components were identified in previous works [9,14], contributing to a total of 84.3% and 77.6% of the *H. coccineum* essential oil composition, respectively. Volatiles such as α-pinene, β-pinene, bornyl acetate, spathulenol, and (*E*)-nerolidol were present in both the samples of the present study (HCCAO and HCCRO) and previous investigations in varying amounts. *p*-cymene, *cis*-linalool oxide, *trans*-linalool oxide, *trans*-pinocarveol, *trans*-verbenol,borneol, α-terpineol, α-himachalene, β-acoradiene, globulol, viridiflorol, γ-eudesmolagarospirol, β-eudesmol, α-cadinol, and bulnesol were present only in HCCRO, whereas Sabinene, myrcene, artemisia alcohol, sulcatone, *cis*-sabinene hydrate, thujol, isoborneol, β-bourbonene, β-cubebene, β-elemene, γ-elemene, α-humulene, 9-*epi*-(*E*)-caryophyllene, α-neocallitropsene, germacrene D, bicyclogermacrene, α-farnesene, naphthalene, β-vetispirene, elemol, 7-hydroxyfarnesen, salvial-4(14)-en-1-one, rosifoliol, ledol, cadin-4-en-10-ol, α-(*Z*)-bergamotol, iso-longifolol, (*E*)-isovalencenol, and dodeca-(2*E,*4*E*)-dienal were found only in HCCAO.

Phytochemicals such as sabinene, myrcene, β-ocimene, γ-terpinene, artemisia alcohol, sulcatone, *cis*-sabinene hydrate, thujol, isoborneol, β-bourbonene, β-cubebene, β-elemene, γ-elemene, α-himachalene, α-humulene, 9-*epi*-(*E*)-caryophyllene, β-acoradiene, α-neocallitropsene, germacrene D, bicyclogermacrene, δ-cadinene, naphthalene, β-vetispirene, β-dihydroagarofuran, kessane, elemol, davanone B, 7-hydroxyfarnesen, salvial-4(14)-en-1-one, guaiol, rosifoliol, ledol, γ-eudesmol, cadin-4-en-10-ol, agarospirol, β-eudesmol, α-(*Z*)-bergamotol, iso-longifolol, *(E)*-isovalencenol, and dodeca-(2*E*,4*E*)-dienal were not previously reported, and were identified only in the samples from the present investigation (plants collected from Kausani, Uttarakhand, India). Similarly, compounds such as *trans*-carveol,*cis*-linalool oxide (furanoid), *trans*-linalool oxide (furanoid), *trans*-pinocarveol, *trans*-verbenol, myrtenol, α-fenchyl acetate, *trans*-sesquisabinene hydrate, caryophyllene oxide, ar-turmerol, and bisabolol identified in the previous study were missing in HCCAO and HCCRO. The results were significant in view of chemo-diversity in *H. coccineum* growing in Himalayan regions and other part of the world. This could be due to variation in their altitudes, environmental circumstance, climatic conditions, geographical distribution, etc.

### 2.2. Principal Component Analysis

**Figure 3:**
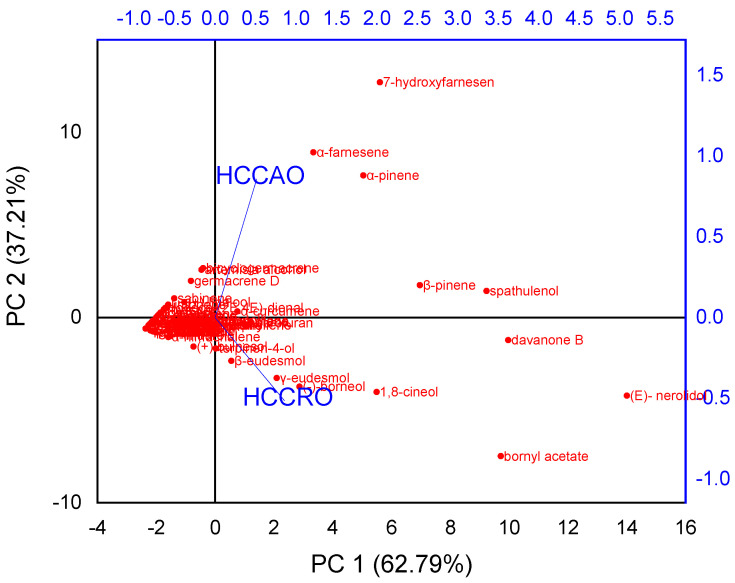
Principal Component Analysis (PCA) of HCCAO and HCCRO.

Principal Component Analysis (PCA) is one of the best multivariate statistical methods used to describe most significant aspects of a dataset. PCA pattern recognition of two essential oils was used to evaluate the phytochemical variability due to the type of plant portion from which essential oils were obtained. The collective contribution rate of variance of the first two principal components (PC1 and PC2) obtained from the PCA method was 100% for chemical compositional differences, which describes most of the variance information. Therefore, these two PCs defined the total compositional variability in the essential oils. PC1 contributed 62.79% in the total variance, which was positively correlated with α-famesene, α-pinene, β-pinene, spathulenol, and 7-hydroxyfamesen, whereas contribution of PC2 to the variance was 37.21%, which was positively correlated with β-eudesmol, γ-eudesmol, 1,8-cineol, davanone B, bornyl acetate, and (*E*)-nerolidol. The Principal Component Analysis (PCA) of HCCAO and HCCRO is shown in Figure 3.

### 2.3. Nematicidal Activity

#### 2.3.1. Effect on Mortality of Second Stage Larvae of *M. incognita*

The nematicidal activity of HCCAO and HCCRO was applied to second-stage juveniles (J_2_) of *M. incognita* for durations of 24, 48, 72, and 96 h. Percent mortality for both the samples was found to increase with an increase, in concentration as well as the incubation time with the essential oils. After 96 h, HCCAO was found to be most effective at 1 µL/mL dose level with 41.33% inhibition in larval mobility, followed by 0.5 µL/mL with 30.66% inhibition. HCCRO was also found to be most effective at 1µL/mL dose level, with 61.66%, inhibition in larval mobility, followed by 0.5 µL/mL with 52.66% inhibition. Silva-Aguayo et al. [19] reported significant nematicidal activity of the essential oil (from *Peumusboldus*) against *Haemonchus contortus* at similar levels of concentration (0.25, 0.5, and 1.0 µL/mL). The overall activity of HCCRO for the durations of 24, 48, 72, and 96 h was observed to be higher than HCCAO. HCCAO and HCCRO exhibited significant variation in immobility against *M. incognita* larvae. The LC_50_ values of the HCCAO at 24, 48, 72, and 96 h after treatment were 0.26, 0.13, 0.06, and 0.003% and LC_50_ values of HCCRO were 2.34, 6.92, 2.33, and 0.23%, respectively. The detailed experimental observation of percentage mortality and LC_50_ values of HCCAO and HCCRO for nematicidal activity against second-stage juveniles of *M. incognita* has been represented in Table 2 and Table 3, respectively.

#### 2.3.2. Effect on Egg Hatchability of *M. incognita*

HCCAO and HCCRO showed a strong inhibitory effect on hatching from eggs in a concentration-dependent manner. The rate of egg hatching was found to be directly proportional to exposure time period and inversely proportional to oil sample concentration. In comparison with HCCAO, HCCRO had a stronger inhibitory effect on *M. incognita* in terms of egg hatching. After 96 h, the maximum rate of egg hatching in HCCAO (55.00%) and HCCRO (22.66%) was observed at a dose level of 0.25 µL/mL, while the minimum rate of egg hatching in HCCAO (17.66%) and HCCRO (11.33%) was observed at 1 µL/mL. Therefore, maximum egg hatching inhibition was observed in HCCRO at lowest as well as highest concentration levels. It was discovered that increasing the concentration of HCCAO and HCCRO delayed the start of egg hatching. The IC_50_ values at 24, 48, 72, and 96 h were 2.18, 2.38, 2.48 and 2.72 µL/mL for HCCAO and 1.92, 2.06, 2.19 and 2.07 µL/mL were for HCCRO respectively. The detailed experimental observations of percent egg hatching and IC_50_ values of HCCAO and HCCRO on the egg hatching of *Meloidogyne incognita* have been represented in Table 4 and Table 5, respectively.

It has been reported that β-dihydroagarofuran, kessane, elemol, (*E*)-nerolidol, davanone B, spathulenol, 7-hydroxyfarnesen, rosifoliol, T-muurolol, linalool, and *E*-isovalencenol were among the most oxygenated sesquiterpenoids observed as main components in plant essential oils, and showed egg-hatching and nematicidal activity in terms of mortality against the root knot nematode, *M. Incognita* [20]. Oxygenated sesquiterpenoid (*E*)-nerolidol, davanone B, spathulenol, 7-hydroxyfarnesen, globulol, and τ-muurolol) have been reported to efficiently inhibit the nematode eggs hatching and mortality, which indicates that essential oils with a high content of these compounds could be useful as natural nematicides for the control of *M. incognita*. The presence of one of the single major compounds or synergetic effects of major and minor constituents of essential oil might be responsible for the nematicidal activity of HCCAO and HCCRO towards the egg hatching and immobility of second-stage larvae of *M. incognita* [21,22].

### 2.4. Insecticidal Activity

The insecticidal activity of essential oils from rhizome and the aerial part of *H. coccineum* was estimated against *Spodoptera litura* (cotton cutworm) insects using the leaf-dip method. Fourth instar larvae of *S. litura* were used for different concentrations of essential oils to test the activity. The experiment was conducted in triplicate, and the total number of test insects per treatment was five. Tween-20 (1.0%) water solution was taken as control. The results showed that HCCRO was more effective than HCCAO and showed good mortality in a concentration-dependent manner (Table 6). During the experiment, no mortality was observed after 72 h. The mortality percentage of *S. litura* insect, treated with the essential oils of rhizome and aerial part of *H. coccineum,* is presented in Table 6. The LC_50_ values of HCCAO were 0.007, 0.006, and 0.005%, and the values of HCCRO were 0.007, 0.006, and 0.005% at 24, 48, and 72 h, respectively. The LC_30_, LC_50_, and LC_90_ value of essential oils from rhizome and the aerial part of *H. coccineum* are presented in Table 7. Significant insecticidal activity was reported for the essential oil (*Mentha pulegium*) at concentrations similar to the present investigation (10–100 µL) in fumigation conditions against *Bruchus rufimanus* [23].

The insecticidal efficacy of *H. coccineum* rhizome essential oil has also been reported against three insects, *Stephanitis pyrioides*, *Aedes aegypti*, and *Solenopsisinvicta* [9]. The toxicity of essential oils against test insect might be due to the presence of various terpenoids found in the essential oils, or even may be due to the interaction of the major and the minor components present in the botanicals.

### 2.5. Herbicidal Activity

#### 2.5.1. Inhibition of Seed Germination

The mean percent seed germination inhibition of essential oils from aerial part and rhizome of *H. coccineum* at different concentrations (50–200 µL/mL) has been depicted in Table 8. The essential oils possess moderate herbicidal activity in a dose-dependent manner. The herbicidal activity of rhizome and aerial part essential oil of *H. coccineum* at the highest concentration (200 µL/mL) was found in the order of HCCRO (96%) > HCCAO (92.00%). Essential oils from *Limnophila indica* have also been reported to have significant herbicidal activity at similar levels of treatment concentrations (50–200 µL/mL) [24]. IC_50_ was calculated at the time when 100% germination was achieved in the control and is used to compare the relative herbicidal activities of all the samples, as the lower the herbicidal activity, the higher its IC_50_ values. The order in which the activity was observed in terms of LC_50_ was as follows: HCCRO (62.78 ± 5.86 µL/mL) > HCCAO (88.09 ± 3.42 µL/mL) in Table 9.

It was observed that HCCRO exhibited more herbicidal activity than HCCAO. Herbicidal activity of the *Hedychium spicatum* rhizome essential oil has also been reported against Radish (*Raphanus raphanistrum*) seeds in a previous study [25]. It was inferred that the herbicidal activity was due to the presence of various bioactive components such as camphor, 1,8-cineole, isoborneol, and linalool in the essential oil, or might be a possible synergistic effect of the minor as well as major compounds present in the *H. coccineum* rhizome and aerial part essential oils.

#### 2.5.2. Inhibition of Root Length

The inhibition of root length was assessed as the measure of herbicidal activity. The percent root length inhibition of seeds germinated was calculated when 100% germination was achieved at various concentration ranges of 50, 100, 150, and 200 µL/mL. In the case of HCCRO, the percent inhibition of root length was recorded as 34.44%, 53.33%, 67.77%, and 84.07% from lowest to highest concentrations, while in the case of HCCAO, the percent inhibition was measured as 27.03%, 56.29%, 73.33%, and 90.37%, respectively, from lower to higher concentrations, as represented in Table 10. IC_50_ was calculated when 100% germination was achieved in the control, and was used to compare the relative herbicidal activities in terms of inhibition of root growth of all the samples, as the lower the herbicidal activity, the higher its IC_50_ values. The order in which the activity was observed was as follows: HCCRO (94.68 ± 2.74 µL/mL) > HCCAO (96.85 ± 0.38 µL/mL) (Table 11).

#### 2.5.3. Inhibition of Shoot Length

The inhibition of shoot length was also assessed as the measure of herbicidal activity. The percent shoot length inhibition was calculated when 100% germination was achieved at various concentrations ranging between 50, 100, 150, and 200 µL/mL. In case of HCCRO, the percent inhibition of shoot length was recorded as 40%, 47.77%, 74.44%, and 99.62% from lowest to highest concentrations, while in case of HCCAO, the percent inhibition was measured as 34.44%, 52.22%, 66.66%, and 81.11%, respectively, from lower to higher concentrations, and represented in Table 12. IC_50_ was calculated when 100% germination was achieved in the control, and was used to compare the relative herbicidal activities in terms of inhibition of root growth of all the samples, as the lower the herbicidal activity, the higher its IC_50_ values. The order in which the activity was observed in terms of IC_50_ values was as follows: HCCRO (87.44 ± 2.98 µL/mL) > HCCAO (133.06 ± 17.22 µL/mL) (Table 13).

### 2.6. Antifungal Activity

The antifungal activity of HCCAO and HCCRO was evaluated against two phytopathogenic fungi (*Fusarium oxysporum* and *Curvularialunata*) at varied doses (50–750 µL/mL). The antifungal activity of the essential oils is shown in Table 14. The essential oils exhibited good antifungal activity by inhibiting the mycelial growth of pathogenic fungi. HCCRO (88.1%) had the maximum antifungal activity against *F. oxysporum,* followed by HCCAO (83.3%), while HCCAO (84.1%), followed by HCCRO (74.8%), had the strongest antifungal activity against *C. lunata* at higher concentrations (750 µL/mL). The antifungal activity of HCCAO and HCCRO was significantly lower compared to standard fungicide Carbendazim (100%), even at a higher concentration (750 µL/mL) against both the tested fungi. Antifungal activity was also demonstrated for the essential oil at 50–500 µL/mL in a previous study [26].

Several biologically active compounds, such as (*E*)-nerolidol, davanone B, spathulenol, limonene, (*E*)-caryophyllene, bicyclogermacrene, and 7-hydroxyfarnesen have been reported to possess the antifungal properties of the essential oils tested against *Colletotrichum acutatum, C. fragariae, and C. gloeosporioides* [9]. Studies have confirmed that the *Hedychium* essential oil, which is rich in (*E*)-nerolidol*,* α-farnesene, α-pinene, and β-pinene, shows potential antifungal activity against *Candida albicans* and *Fusarium oxysporum* [27]. The presence of individual major compounds or the synergetic effect of major/minor constituents of essential oil might be responsible for the antifungal activity of HCCAO and HCCRO towards *F. oxysporum* and *C. lunata*.

### 2.7. Antibacterial Activity

The emerging antibiotic resistance in bacteria and the high cost of developing novel antimicrobial drugs has encouraged researchers to search for novel effective and economically viable broad-spectrum natural products with different modes of action. Essential oils and their chemical constituents in pure form have been reported to have effective action against resistant microbial strains [28,29,30]. Therefore, in this study, we have explored the antibacterial activity of HCCRO and HCCAO using zones of inhibition assay against Gram-positive bacteria, *Staphylococcus aureus,* and Gram-negative bacteria, *Salmonella enterica* serovar Typhi. The spot diffusion method confirmed that both HCCAO and HCCRO showed antibacterial activity against both the bacterial pathogens. However, HCCRO showed a higher zone of inhibition against both Gram-positive and Gram-negative pathogens. Of these strains, Gram-positive *Staphylococcus aureus* was more susceptible to HCCRO than Gram-negative *Salmonella enterica* serovar Typhi*,* with average zones of inhibition of 25 mm and 6 mm, respectively. *Staphylococcus aureus* is a Gram-positive opportunistic pathogenic bacterium which causes nosocomial and community infections such as bloodstream infections, pneumonia, skin and soft tissue infections, and bone and joint infections [31]. *Salmonella enterica* serovar Typhi is a common and clinically significant Gram-negative pathogenic bacterium that causes gastroenteritis and typhoid fever in humans, affecting over 20 million people worldwide and killing 220,000 people each year [32,33]. Results showed that HCCRO had potential antibacterial activity against both bacterial pathogens. The colony farming unit (CFL/mL) of *Staphylococcus aureus* and *Salmonella enterica* serovar Typhi by essential oils from the aerial and rhizome part of *H. coccineum* is represented in Table 15.

#### Determination of Minimum Inhibitory (MIC) Concentration and Minimum Bactericidal Concentration (MBC)

The minimal inhibitory concentration (MIC) and minimum bactericidal concentration (MBC) values were determined using the broth dilution method to evaluate the effectiveness in controlling bacterial pathogens. The results revealed that in the presence of HCCRO (2.5 μL/100 μL) and HCCAO (2.5 μL/100 μL), 6.5 and 6 Log CFU/mL, respectively, reductions in the growth of *Staphylococcus aureus* were observed, while the growth was completely inhibited at higher concentration (5 μL/100 μL). The MIC and MBC values of HCCRO against *Staphylococcus aureus* were 2.5 μL/100 μL and 5 μL/100 μL, respectively. Meanwhile, in the case of *Salmonella enterica* serovar typhi, 3 and 2.3 log reductions in the CFU were observed in the presence of HCCRO and HCCAO, respectively. Changes in bacterial cell suppression by essential oils could be attributed to chemical components and the volatile nature of their components, or differences in the composition of Gram-positive and Gram-negative bacterial membranes [34,35].

It has been observed that HCCRO exhibits more antibacterial efficacy than HCCAO against *Staphylococcus aureus* and *Salmonella enterica serovar Typhi*. Studies have confirmed that the essential oil rich in α-farnesene, α-pinene and (*E*)-nerolidol shows potential antibacterial activity against *Staphylococcus aureus*, *Penicillium chrysogenum*, *Bacillus subtilis, Escherichia coli,* and *Saccharomyces cerevisiae* [36]. The essential oils of *H. venustum*, *H. spicatum*, *H. coronarium,* and *H. flavescens* have also been reported for their antibacterial activity against *Salmonella typhi* and *Escherichia coli* [37]. Antibacterial efficacy of HCCAO and HCCRO might be due to the presence of the main constituents such as 7-hydroxyfarnesen, bicyclogermacrene, germacrene D, α-farnesene, (*E*)-caryophyllene, α-farnesene (11.1%), α-pinene (10.9%), (*E*)-nerolidol (15.9%), bornyl acetate (13.9%), davanone B (10.9%), and spathulenol (8.9%), or might be a possible synergistic effect of the major/minor compounds present in the *H. coccineum* rhizome and aerial part essential oils.

### 2.8. In Silico PASS Prediction of HCCAO and HCCRO

In silico PASS predictions for antibacterial, antifungal, and nematicidal activity of selected phytochemical compounds from HCCAO and HCCRO are reported in Table 16. Among the identified compounds, davanone B, α-farnesene, davanone B, α-curcumene, germacrene D, and (*E*)-caryophyllene were observed to exhibit acceptable Pa/Pi values. However, other compounds were observed to exhibit negligible nematicidal activity as per PASS prediction. These data support the in vitro nematicidal activity for HCCRO and HCCAO performed in the present investigation. From the PASS prediction data, it can be inferred that the nematicidal activity of these essential oils is governed by one of the above-mentioned compounds having acceptable Pa/Pi values or the result of the synergistic effect of more than one component present in essential oil. Volatile compounds exhibited a good Pa/Pi range, (0.45 > 0.02). Among the identified compounds, 7-hydroxyfarnesen, bicyclogermacrene, germacrene D, *α*-farnesene, (*E*)-caryophyllene, and (*E*)-nerolidol were found to exhibit acceptable antibacterial effects (in terms of Pa/Pi values). However, some other major compounds, such as β-pinene, 1,8-cineol, borneol, γ-eudesmol, α-curcumene, and β-dihydroagarofuran were predicted to have comparatively low antibacterial activities. Overall, the PASS prediction supported the antibacterial activity of HCCAO and HCCRO compounds. The Pa/Pi value of major compounds such as (*E*)-nerolidol, linalool, α-farnesene, davanone B,limonene, (*E*)-caryophyllene, bicyclogermacrene, 7-hydroxyfarnesen, and spathulenol for the antifungal potential was higher than that of these compounds for antibacterial activity. The other predicted compounds also exhibited superior antifungal activity. Hence, the PASS prediction supports the present high antifungal activities of HCCAO and HCCRO. Therefore, it is supposed that these biological activities of HCCAO and HCCRO are governed by the compounds showing a higher Pa/Pi ratio, or it may be a combined effect of more than one compound.

## 3. Materials and Methods

### 3.1. Plant Material

*H. coccineum* plant material was collected in August 2021 from Kausani (Altitude-1672 m, Latitude 29.8445° N, and Longitude 79.6039° E), Bageshwar, Uttarakhand, India. Dr. D.S. Rawat (Plant Taxonomist), Department of Biological Sciences, College of Basic Science and Humanities, G.B.P.U.A.T, Pantnagar, recognized the plant material and submitted the herbarium (specimen no. GBPUH-1040) to the Department of Biological Sciences.

### 3.2. Essential Oil Isolation

The essential oils from the aerial part and rhizome of *H. coccineum* were extracted using the hydro distillation method by subjecting the fresh plant materials (1.2 kg of arial part and 0.9 kg rhizome) to the Clevenger-type apparatus for about 3 h [38,39,40]. The obtained essential oils were dried over anhydrous sodium sulphate before being filtered and stored in dark glass vials at 4 °C for further use.

### 3.3. GC-MS Analysis

The phytochemical composition of both essential oils was analyzed using gas chromatography-mass spectrometry (GC-MS) analysis (A.I.R.F. (J.N.U), New Delhi, India) with a GCMS-QP 2010 Ultra DB-5 and GCMS-QP 2010 Ultra Rtx-5MS column (30 m × 0.25 mm i.d., 0.25 µm). Helium was employed as a carrier gas at a flow rate of 1.21 mL/min, with a split ratio of 10.0. The GC oven temperature program was 50–280 °C with a temperature gradient of 3 °C/min up to 210 °C (isotherm for 2 min), then 6 °C/min up to 280 °C. The constituents of essential oils were identified by comparing their mass spectrum fragmentation patterns and their relative retention index (RI) values with the MS library (NIST14.lib, FFNSC2.lib, WILEY8.LIB), as well as comparing the spectra with literature data [15].

### 3.4. Nematicidal Activity

#### 3.4.1. Nematode Population Collection

*Meloidogyne incognita* eggs were collected from nematode-infected tomato (*Solanum lycopersicum*) roots collected from the Crop Research Center, G. B. P.U.A.T, Pantnagar, in a glasshouse, maintained at 25 ± 2 °C. The sample was collected on the basis of the visual symptoms of root knots or galls formed in the plant. Hand-picked matured egg masses from infected tomato roots were cultured in distilled water in a growth chamber at 25 °C. For future use, emerged juveniles were collected and preserved at 5 °C [41,42].

##### In Vitro Mortality Assay on Second Stage Larvae of *M. incognita*

For in vitro mortality assay, second-stage juveniles (100 in number) collected from hatched eggs within 48 h were placed on gridded Petri dishes with stock solution and 1.0 mL of distilled water. There were three different doses, i.e., 0.25, 0.5, and 1 µL/mL of essential oils in a 1.0% Tween-20 water solution. The treatments were performed in triplicate and arranged in randomized order. The juveniles immersed in Tween-20 (1.0%) water solution were used as a control group. The number of dead juveniles was counted using a stereo-binocular microscope throughout time periods of 24, 48, 72, and 96 h. Totally motionless (dead larvae) nematodes were picked out of the Petri dish and placed in distilled water. Percent mortality was calculated using Abbott’s formula [43].

$$\left(\%\right)\;\mathrm{motality}=\left(\mathrm{Nt}-\frac{\mathrm{Nc}}{100}-\mathrm{Nc}\right)\times 100$$

where, Nt = Mortality in treatment; Nc = Mortality in control.

##### Effect of Essential Oils on Egg Hatchability Test of *M. incognita*

Two egg masses of *M. incognita* were suspended in 0.25, 0.5, and 1 µL/mL conc. of HCCAO and HCCRO in gridded Petri dishes. The egg masses suspended in a Tween-20 (1.0%) water solution were used as a control. All of the treatments were set up in triplicate and in a completely random order in the BOD incubator at a constant temperature of 27 ± 1 °C. Observations on percent egg hatching were made at time intervals of 24, 48, 72, and 96 h. The counting of the number of eggs hatched was performed under a microscope at a magnification of 4×. Percent egg hatching was computed using Abbott’s formula [44].

(1) $$\left(\%\right)\;\mathrm{egg}\;\mathrm{hatching}\;=\left(\mathrm{Nt}-\frac{\mathrm{Nc}}{100}-\mathrm{Nc}\right)\times 100$$

where, Nt = egg hatching in treatment; N_C_ = egg hatching in control.

### 3.5. Insecticidal Activity

#### 3.5.1. Test Insect

Insecticidal activity of HCCAO and HCCRO were tested against cotton cut worm (*Spodoptera litura* belongs to family: Noctuidae and order: Lapidoptera), which is a serious polyphagous pest in Asia, Oceania, and the Indian subcontinent. Although it is a harmful pest in tobacco, it also attacks cole crops, castor, cotton, chilli peppers, tomato, etc.

#### 3.5.2. Collection of Larvae and Maintenance

Initial culture of *S. litura* as egg mass was collected from wild castor (*Ricinus communis*) plant from CRC (Crop Research Center), G.B.P.U.A&T., Pantnagar, Uttarakhand, India. The test insects were reared in a clean plastic container covered with muslin cloth in ideal laboratory conditions, with the temperature kept at 27 °C, and humidity kept at 75–80%. Test insects were served fresh castor leaf every day until they reached the fourth instar larval stage. Finally, fourth instar larvae were starved for 12 to 24 h before being used in insecticidal activity.

#### 3.5.3. Bioassay of Insecticidal Activity

The leaf dip method was used to assess the insecticidal activity of rhizome and aerial part essential oils of *H. coccineum* [45]. For evaluating the insecticidal activity, different concentrations of essential oils (10, 25, 50 and 100 µL/mL) were prepared in Tween-20 (1.0%) solution in distilled water. The castor leaves were cleaned and washed in distilled water before being air dried for an hour. Each castor leaf was sliced into a 25 sq.cm section and immersed in various concentrations of essential oils. The leaf discs were slanted on blotting paper for 2–3 min before being placed in the tray to drain excess solution for 2 h at room temperature. Four instar adult five larvae were released in individual Petri dishes after being starved for 12–24 h. Blotting paper was placed at the bottom of each plate. For 72 h, these Petri plates were monitored for any insecticidal activity. This activity took place in ideal laboratory conditions, with a temperature of 27 °C and a relative humidity of 75–80%. The mortality (%) was calculated after 24, 48, and 72 h of the treatment using Abbott’s formula [43]. LC_50_ values were analyzed using Probit analysis [46].

$$\left(\%\right)\mathrm{Mortality}=\left(T-\frac{C}{100\;\mathrm{of}\;\mathrm{initial}\;\mathrm{populations}-C}\right)\times 100$$

where, T = Mortality in treatment; C = Mortality in control.

### 3.6. Herbicidal Activity

#### 3.6.1. Evaluation of Herbicidal Activity

The herbicidal action of essential oils was assessed based on various parameters such as inhibition of seed germination, inhibition of shoot length, and inhibition of root length against *R. raphanistrum* subsp. *Sativus* (Radish) seeds.

#### 3.6.2. Herbicidal Bioassay

The herbicidal activity of essential oils was evaluated using the method reported by [47,48,49,50]. *Raphanus raphanistrum* subsp. *Sativus* (L.) (Radish) seeds were obtained from the VRC (Vegetable Research Centre), G.B.P.U.A.T. Pantnagar. To evaluate the seed germination inhibition, various conc. of essential oils were prepared in Tween-20 (1.0%) aqueous solution. Prior to usage, *R. raphanistrum* subsp. *sativus* seeds were surface sterilized for 15 min in a 5% sodium hypochlorite solution. Ten sterilized seeds *of R. raphanistrum* sub sp. *sativus* were placed on the Petri plates, which were coated with regular filter papers. Then, 2 mL of various concentrations of the tested sample were put onto the plates and left to germinate at 25 ± 1 °C for 12 h in an incubator. Pendimethalin was used as a standard herbicide. Tween-20 (1.0%) solution in sterilized distilled water was taken as a control for essential oils. Percent inhibition of seed germination and inhibition of root and shoot length were measured after 5 days of incubation. The formulae used for determination of inhibition of seed germination, inhibition of shoot length, and inhibition of root length are as follows.

- Inhibition of seed germination

$$\left(\%\right)\;\mathrm{Inhibition}\;\mathrm{of}\;\mathrm{seed}\;\mathrm{germination}=100\times \left(1-\frac{\mathrm{Gt}}{\mathrm{Gc}}\right)$$

where, Gt—no. of seeds germination in treatment;

Gc—No. of seeds germination in control.

b. Inhibition of shoot length

$$\left(\%\right)\;\mathrm{Inhibition}\;\mathrm{of}\;\mathrm{shoot}\;\mathrm{length}=100\times \left(1-\frac{\mathrm{Ct}}{\mathrm{Cc}}\right)$$

where, Ct –shoot length in treatment;

Cc—shoot length in control.

c. Inhibition of root length

$$\left(\%\right)\;\mathrm{Inhibition}\;\mathrm{of}\;\mathrm{shoot}\;\mathrm{length}=100\times \left(1-\frac{\mathrm{Rt}}{\mathrm{Rc}}\right)$$

where, Rt—root length in treatment;

Rc—root length in control.

### 3.7. Antifungal Activity

*Fusarium oxysporum* and *Curvularia lunata*, two phytopathogenic fungi, were provided by the Department of Plant Pathology, College of Agriculture, G.B.P.U.A.T, Pantnagar, India. HCCRO and HCCAO were tested against the test fungus using the poisoned food technique developed by [51]. The phytopathogenic fungi were revived and grown by placing the fungal colonies aseptically on the Petri plates containing the Potato Dextrose Agar (PDA) media. The Petri plates were incubated for one week at 26 ± 2 °C. The assay discs (diameter = 5 mm) of a 7-day-old culture of the test fungus were inoculated aseptically, with the prepared plates containing varied conc. of essential oils (50–750 µL/mL) prepared in Tween-20 (1.0%) water solution. A control devoid of essential oils was prepared under the same conditions. The control plate was cultured for 7 days until the growth reached the plate’s edge. The percent inhibition of radial growth of each fungal strain was calculated in comparison with the control. Antifungal activity was detected by clear zones of mycelia growth inhibition surrounding the Petri plate, which were measured in millimeters. Carbendazim (50% WP) was employed as the standard fungicide, and percent inhibition was calculated using McKinney’s formula [46].

$$\left(\%\right)\;\mathrm{Inhibition}\;=\left(X-\frac{Y}{X}\right)\times 100$$

where, X = Radial growth in control, Y = Radial growth in treatment.

### 3.8. Antibacterial Activity

#### 3.8.1. Diffusion Agar Antibacterial Assay

The antibacterial activity of the essential oils was investigated qualitatively via diffusion assay. Briefly, the overnight grown bacterial cultures (*Staphylococcus aureus* and *Salmonella enterica* serovar typhi) were sub-cultured in Luria Bertani (LB) broth and grown till OD_600nm_ reached 0.2. Next, 100 µL of the above culture of each bacterial cell was spread plated on an LB agar plate. Then, 10 µL of rhizome and aerial essential oils was spotted onto the LB agar plates separately and incubated at 37 °C for 24 h. Upon incubation, the inhibition zone diameter of the inoculated plate was measured.

#### 3.8.2. Determination of Minimum Inhibitory Concentration

The susceptibility of both Gram-positive (*Staphylococcus aureus*) and Gram-negative (*Salmonella enterica* serovar Typhi) bacterial cells to essential oils was estimated by the micro broth dilution method as per clinical and laboratory standards institute (CLSI) guidelines in brain heart infusion (BHI) and MH broth, respectively [52,53,54]. Briefly, the overnight grown bacterial cells were sub-cultured in respective broths and grown till the mid log phase (OD reached 0.4). After that, each bacterial cell suspension was diluted 1000-fold to attain an inoculum of 10^5^ colony forming units (10^5^CFU/100 μL) and mixed with an equal volume (100 μL:100 μL) of 2-fold-diluted essential oils. The growth of bacterial cells was assessed by enumerating CFU in the agar plate after incubating the bacterial cells for 12 h under a static condition in a humidity-controlled incubator at 37 °C. The MIC of a plant extract is the lowest concentration that inhibits observable microorganism growth. The experiments were repeated three times, with two replicates in each dish.

### 3.9. In Silico PASS Prediction of Biological Activities

The biological activities of 20 major compounds present in the HCCAO and HCCRO essential oils were predicted using PASS (prediction of activity spectra for substances) software [55,56]. PASS is a free online cheminformatic software that assesses the biological activities of chemical compounds based on structural similarities to a large library of active compounds. Pa or Pi readings were used to calculate the bioactivity score. If the Pa value (chances to be active) was greater than the Pi value (chances to be inactive), the projected compound was likely to be active. HCCAO and HCCRO were predicted to exhibit diverse bioactivities (Pa > Pi).

### 3.10. Statistical Analysis

All of the experiments were carried out in three replicates, with the results represented as mean ± standard deviation (SD). A two-way analysis of variance (ANOVA) followed by a Tukey’s multiple comparison test was performed to test the differences in the means of treatment using RStudio2021.09.2. OriginPro 2021 version 9.8.0.200 was used to perform Principal Component Analysis (PCA) on the chemical composition of the essential oils under investigation to identify the most significant feature in the dataset.

## 4. Conclusions

According to the present study, it can be observed that GC-MS analysis of aerial part and rhizome essential oils (HCCAO and HCCRO) of *H. coccineum* showed the presence of 50 and 32 compounds, respectively. The tested essential oils possessed significant antibacterial (*S. aureus* and *S. typhi*) and antifungal (against *F. oxysporum and C. lunata*) activities and moderate nematicidal (against *M. Incognita*), insecticidal (against *S. litura),* and herbicidal (against *R. raphanistrum* subsp. *sativus*) activity in a tested concentration, which can be used to create a highly effective botanical pesticide. The antimicrobial action of *H. coccineum* essential oil on bacterial and fungal strains demonstrated the plants’ potential as a source of natural antimicrobial agents. Nematicidal activity of the essential oils might be a good source of more selective, biodegradable, and environmentally friendly natural nematicides, acting as a substitute to synthetic nematicides and a good source of herbal nutraceuticals and phytochemicals. The herbicidal activity results were also validated by IC_50_ values, as the higher the IC_50_ value, the lower the herbicidal activity. The order in which the samples exhibited herbicidal potential in terms of percent seed germination inhibition was found HCCRO (62.78 ± 5.86 µL/mL) > HCCAO (88.09 ± 3.42 µL/mL). Herbicidal potential in terms of root length inhibition was found in the following order: HCCRO (94.68 ± 2.74 µL/mL) > HCCAO (96.85 ± 0.38 µL/mL), while herbicidal potential in terms of shoot length inhibition was found in the following order: HCCAO (133.06 ± 17.22 µL/mL) > HCCAO (87.44 ± 2.98 µL/mL), respectively.

## Figures and Tables

**Table 1:** Comparative chemical composition of HCCAO and HCCRO with previous work.

S.N.	Compound Identified	RI^c^	RI^L^	Composition % in Present Study (Kausani, Kumaun Region, Uttarakhand, India)		% Composition of Rhizome Essential Oils in Reported Studies	
				HCCAO	HCCRO	Sakhanokho et al. [9] (Mississippi, U.S.)	Gurib-Fakim et al. [14]. (Pamplemousses, Mauritius)
1.	Artemisia alcohol	939	935	3.7	-	-	-
2.	α-pinene	948	939	10.9	2.1	13.5	2.4
3.	Camphene	953	954	0.1	0.1	2.3	-
4.	Sulcatone	968	965	0.7	-	-	-
5.	Sabinene	972	975	1.9	-	-	-
6.	β-pinene	978	979	6.8	6.8	7.5	1.8
7.	Myrcene	991	990	1.2	-	-	-
8.	*p*-cymene	1024	1024	-	0.2	0.5	-
9.	Limonene	1030	1029	1.5	-	1.1	0.2
10.	1,8-cineol	1032	1031	1.1	8.5	0.1	-
11.	β-ocimene	1046	1050	0.4	-	-	-
12.	γ-terpinene	1058	1059	t	0.3	-	-
13.	*Cis*-sabinene hydrate	1069	1070	t	-	-	-
14.	*Cis*-linalool oxide (Furanoid)	1082	1072	-	-	2.0	-
15.	*Trans*-linalool oxide (Furanoid)	1088	1086	-	-	1.8	-
16.	Linalool	1092	1096	1.9	0.4	26.7	-
17.	Thujol	1098	1095	0.3	-	-	-
18.	*Trans*-pinocarveol	1139	1139	-	-	1.5	-
19.	*Trans*- verbenol	1143	1144	-	-	0.9	-
20.	Camphor	1149	1146	0.5	0.6	0.2	-
21.	Isoborneol	1165	1160	0.5	-	-	-
22.	Borneol	1169	1169	-	6.1	1.0	-
23.	Terpinen-4-ol	1177	1178	0.3	2.6	0.1	-
24.	α-terpineol	1183	1188	-	0.4	0.6	0.6
25.	Naphthalene	1189	1181	t	-	-	-
26.	Myrtenol	1194	1195	-	-	1.2	-
27.	Trans-carveol	1217	1216	-	-	0.3	-
28.	α-fenchyl acetate	1223	1220	-	-	-	0.2
29.	Bornyl acetate	1285	1285	0.3	13.9	8.4	0.8
30.	β-elemene	1390	1390	0.3	-	-	-
31.	β-cubebene	1392	1389	t	-	-	-
32.	β-bourbonene	1393	1388	0.1	-	-	-
33.	α-*cis*-bergamotene	1416	1412	0.8	0.6	-	-
34.	(*E*)-caryophyllene	1424	1419	0.9	0.7	1.5	-
35.	γ-elemene	1432	1436	0.8	-	-	-
36.	α-himachalene	1449	1451	-	0.9	-	-
37.	α-humulene	1461	1454	0.9	-	-	-
38.	9-epi-(*E*)-caryophyllene	1464	1466	0.2	-	-	-
39.	β-acoradiene	1471	1470	-	0.3	-	-
40.	α-curcumene	1479	1480	2.4	2.2	4.1	-
41.	α-neocallitropsene	1480	1476	0.2	-	-	-
42.	Germacrene D	1480	1481	3.0	-	-	-
43.	β-vetispirene	1494	1493	0.4	-	-	-
44.	Bicyclogermacrene	1497	1500	3.8	-	-	-
45.	α-farnesene	1502	1505	11.1	-	-	1.9
46.	β-dihydroagarofuran	1509	1503	1.0	1.1	-	-
47.	δ-cadinene	1518	1523	0.5	0.2	-	-
48.	Kessane	1533	1530	0.3	0.7	-	-
49.	Elemol	1551	1549	0.5	-	-	-
50.	(*E*)-nerolidol	1564	1563	5.3	15.9	4.6	44.4
51.	Davanone B	1567	1566	5.8	10.9	-	-
52.	Spathulenol	1576	1578	7.7	8.9	3.1	0.4
53.	7-hydroxyfarnesen	1579	1581	15.5	-	-	-
54.	*Trans*-sesquisabinene hydrate	1580	1579	-	-	-	24.2
55.	γ-turmerol	1581	1582	-	-	0.2	-
56.	Caryophyllene oxide	1582	1583	-	-	1.5	-
57.	Globulol	1589	1590	-	0.4	0.8	-
58.	Viridiflorol	1592	1592	-	0.3	0.5	-
59.	Salvial-4(14)-en-1-one	1596	1594	t	-	-	-
60.	Rosifoliol	1598	1601	1.0	-	-	-
61.	Ledol	1600	1602	t	-	-	-
62.	Guaiol	1603	1600	0.5	0.9	-	-
63.	γ-eudesmol	1632	1632	-	5.2	-	-
64.	T-muurolol	1645	1646	0.1	0.9	0.1	-
65.	α-cadinol	1648	1654	-	0.4	0.4	-
66.	Cadin-4-en-10-ol	1649	1647	0.3	-	-	-
67.	Agarospirol	1649	1648	-	0.4	-	-
68.	β-eudesmol	1652	1650	-	3.4	-	-
69.	Bulnesol	1673	1671	-	1.9	-	0.4
70.	α-bisabolol	1686	1685	-	-	-	0.3
71.	α-(*Z*)-bergamotol	1688	1690	0.2	-	-	-
72.	Iso-longifolol	1725	1729	t	-	-	-
73.	(*E*)-isovalencenol	1796	1793	0.5	-	-	-
	Class composition			% Composition			
	Monoterpene hydrocarbons			22.8	9.5	25.2	4.4
	Oxygenated monoterpenes			9.3	32.5	44.5	1.6
	Sesquiterpenes hydrocarbons			25.0	4.9	5.6	1.9
	Oxygenated sesquiterpenes			39.1	51.3	9.0	69.7
	Total (%)			96.2	98.2	84.3	77.6

HCCAO—*Hedychium coccineum* aerial part essential oil; HCCRO—*Hedychium coccineum* rhizome essential oil; “-“—not detected t—trace < 0.1%. RI^c^—Calculated retention indices value; RI^L^—Literature retention indices value on a DB-5 MS column in reference Adams, [15].

**Table 2:** Effect of essential oils on second-stage juveniles (J_2_) of *M. incognita* at different concentration.

Treatment (T)	Concentration. (µL/mL)	Percent Mortality and Exposure Time (h.)			
		24 h	48 h	72 h	96 h
HCCAO	0.25	17.66 ± 0.57 ^no^	21.00 ± 1.00 ^mn^	26.66 ± 1.52 ^ijk^	29.66 ± 1.52 ^hij^
	0.5	24.33 ± 0.57 ^lm^	24.66 ± 0.57 ^lm^	28.66 ± 1.15 ^jkl^	30.66 ± 1.15 ^hi^
	1	25.66 ± 0.57 ^kl^	27.33 ± 1.15 ^ijkl^	33.33 ± 1.52 ^gh^	41.33 ± 1.15 ^e^
HCCRO	0.25	21.00 ± 1.00 ^hi^	30.66 ± 1.15 ^hi^	34.66 ± 0.57 ^g^	46.66 ± 1.52 ^d^
	0.5	30.66 ± 1.15 ^mn^	40.00 ± 1.00 ^ef^	49.00 ± 1.00 ^cd^	52.66 ± 1.15 ^bc^
	1	36.33 ± 1.52 ^fg^	46.33 ± 1.52 ^d^	55.00 ± 1.00 ^b^	61.66 ± 1.52 ^a^
Control	water	1.66 ± 2.08 ^rs^	3.33 ± 1.52 ^rs^	6.33 ± 1.52 ^qrs^	14.33 ± 2.08 ^op^

HCCAO—*H. coccineum* a aerial part essential oil; HCCRO—*H. coccineum* rhizome part essential oil; SD—standard deviation. Within a column, mean values followed by the same letter are not significantly different according to Tukey’s test (*p* < 0.05).

**Table 3:** LC_50_ values of HCCAO and HCCRO for nematicidal activity against second-stage juveniles (J_2_) of *M. incognita*.

Sample	H.	*LC_50_ (%)	Regression Equation.
HCCAO	24	0.26	y = 0.007x + 4.06
	48	0.13	y = 0.006x + 4.39
	72	0.06	y = 0.008x + 4.49
	96	0.03	y = 0.005x + 4.79
HCCRO	24	2.34	y = 0.004x + 4.01
	48	6.92	y = 0.003x + 4.14
	72	2.33	y = 0.003x + 4.29
	96	0.23	y = 0.004x + 4.40

*LC_50_—lethal concentration; HCCAO—*Hedychium coccineum* aerial part essential oil; HCCRO—*Hedychium coccineum* rhizome part essential oil; Reg. eq.—regression equation.

**Table 4:** Nematicidal activity of HCCAO and HCCRO on the egg hatching of *Meloidogyne incognita*.

Treatment (T)	Concentration (µL/mL)	Percent Egg Hatching of Nematodes and Exposure Time (h)			
		24 h	48 h	72 h	96 h
HCCAO	0.25	32.33 ± 0.57 ^j^	43.33 ± 1.52 ^hi^	45.00 ± 1.73 ^gh^	55.00 ± 3.00 ^f^
	0.5	25.00 ± 2.64 ^klm^	30.00 ± 1.00 ^jk^	30.00 ± 2.64 ^jk^	38.33 ± 0.57 ^i^
	1	6.66 ± 2.30 ^t^	14.00 ± 1.73 q^rs^	14.00 ± 2.00 ^qrs^	17.66 ± 2.51 ^opq^
HCCRO	0.25	18.33 ± 0.57 ^nopq^	23.66 ± 1.52 ^lmn^	26.66 ± 0.57 ^kl^	22.66 ± 1.52 ^lmno^
	0.5	9.00 ± 1.00 ^st^	18.00 ± 1.00 ^opq^	19.66 ± 0.57 ^mnop^	15.33 ± 5.70 ^opq^
	1	6.66 ± 1.52 ^t^	11.13 ± 1.48 ^rst^	15.97 ± 1.13 ^pqr^	11.33 ± 0.57 ^rst^
Control	Water	56.33 ± 4.04 ^def^	66.33 ± 2.08 ^cde^	74.33 ± 2.08 ^b^	92.33 ± 3.21 ^a^

HCCAO—*H. coccineum* aerial part essential oil; HCCRO—*H. coccineum* rhizome essential oil; SD—standard deviation. Within the dataset, mean values with same letter in superscript are not significantly different based on Tukey’s test (*p* < 0.05).

**Table 5:** IC_50_ values of HCCAO and HCCRO on the egg hatching of *Meloidogyne incognita*.

Sample	Time (h)	IC_50_ (µL/mL)
HCCAO	24	2.18
	48	2.38
	72	2.48
	96	2.72
HCCRO	24	1.92
	48	2.06
	72	2.19
	96	2.07

HCCAO—*H. coccineum* aerial part essential oil; HCCRO—*H. coccineum* rhizome part essential oil; IC_50_—half maximal inhibitory concentration.

**Table 6:** Mortality percentage of *S. litura* insect treated with HCCRO and HCCAO.

Essential Oil	Concentration (µL/mL)	No. of Insects Used	No. of Insects Dead			% of Average Mortality		
			24 h	48 h	72 h	24 h	48 h	72 h
HCCAO	10	5	0.00 ± 0.00 ^d^	0.00 ± 0.00 ^d^	0.00 ± 0.00 ^d^	0.00	0.00	0.00
	25	5	1.00 ± 0.00 ^cd^	1.66 ± 0.57 ^bc^	2.00 ± 1.00 ^bc^	20.00	33.33	40.00
	50	5	1.66 ± 0.57 ^bc^	2.33 ± 1.57 ^abc^	3.00 ± 0.00 ^ab^	33.33	46.66	60.00
	100	5	2.33 ± 0.57 ^abc^	2.66 ± 0.57 ^ab^	3.66 ± 0.57 ^a^	46.66	53.33	73.33
HCCRO	10	5	0.00 ± 0.00 ^d^	0.00 ± 0.00 ^d^	0.00 ± 0.00 ^d^	0.00	0.00	0.00
	25	5	0.66 ± 0.57 ^cd^	1.33 ± 0.57 ^bcd^	1.66 ± 0.57 ^abc^	13.33	26.66	33.33
	50	5	1.66 ± 0.57 ^bcd^	2.00 ± 1.00 ^abcd^	3.00 ± 1.00 ^ab^	33.33	40.00	60.00
	100	5	2.00 ± 1.00 ^abcd^	2.66 ± 0.57 ^abc^	4.00 ± 0.00 ^a^	40.00	53.33	80.00
Control	water	5	0.00 ± 0.00	0.00 ± 0.00	0.00 ± 0.00	0.00	0.00	0.00

HCCAO—*H. coccineum* aerial part essential oil; HCCRO—*H. coccineum* rhizome part essential oil; SD—standard deviation. Within the dataset, mean values with same letter in superscript are not significantly different, based on Tukey’s test (*p* < 0.05).

**Table 7:** LC_30_, LC_50_, and LC_90_ value for insecticidal activity of HCCRO and HCCAO against *S. litura*.

Sample	Time (h)	LC_30_ (%)	LC_50_ (%)	LC_90_ (%)	Chi-Squared	Regression Equation
HCCAO	24	0.005	0.007	0.01	0.80	y = 0.06x + 0.38
	48	0.004	0.006	0.01	0.83	y = 0.06x + 0.51
	72	0.004	0.005	0.009	0.88	y = 0.06x + 0.43
HCCRO	24	0.006	0.007	0.01	0.82	y = 0.005x + 0.30
	48	0.005	0.006	0.01	0.74	y = 0.062x + 0.42
	72	0.004	0.005	0.008	0.80	y = 0.07x + 0.27

LC—Lethal concentration; HCCAO—*Hedychium coccineum* aerial part essential oil; HCCRO—*Hedychium coccineum* rhizome part essential oil.

**Table 8:** Mean percent seed germination inhibition of HCCAO and HCCRO.

S. No.	Sample Name	% Inhibition of Seed Germination			
	Essential Oil	50 µL/mL	100 µL/mL	150 µL/mL	200 µL/mL
1.	HCCAO	36.00 ± 2.00 ^ab^	51.66 ± 0.57 ^c^	78.66 ± 1.52 ^fg^	92.00 ± 2.00 ^h^
2.	HCCRO	47.66 ± 2.51 ^b^	61 ± 1.00 ^d^	73.33 ± 2.08 ^ef^	96.33 ± 1.52 ^h^
3.	Pendimethalin *	100 ± 0.00	100 ± 0.00	100 ± 0.00	100 ± 0.00

*—Standard herbicide; HCCAO—*Hedychium coccineum* aerial part essential oil; HCCRO—*Hedychium coccineum* rhizome part essential oil; values are means of three replicates ± SD; SD—standard deviation. Within a column, mean values followed by the same letter are not significantly different according to Tukey’s test (*p* < 0.05).

**Table 9:** IC_50_ values of seed germination inhibition of HCCAO and HCCRO.

S. No.	Sample Name	IC_50_ Values (µL/mL) in Triplicates			Mean IC_50_ Values (µL/mL) ± SD
	Essential Oil	I	II	III	
1	HCCAO	90.54	84.18	89.55	88.09 ± 3.42
2	HCCRO	57.29	62.10	68.96	62.78 ± 5.86

HCCAO—*Hedychium coccineum* aerial part essential oil; HCCRO—*Hedychium coccineum* rhizome part essential oil; IC_50_—half maximal inhibitory concentration.

**Table 10:** Mean percent inhibition of root length of HCCAO and HCCRO.

S. No.	Sample Name	% Inhibition of Root Length			
	Essential Oil	50 µL/mL	100 µL/mL	150 µL/mL	200 µL/mL
1.	HCCAO	27.03 ± 0.64 ^a^	56.29 ± 0.64 ^d^	73.33 ± 1.11 ^f^	90.37 ± 0.64 ^h^
2.	HCCRO	34.44 ± 1.11 ^a^	53.33 ± 1.11 ^d^	67.77 ± 1.11 ^e^	84.07 ± 0.64 ^g^
3.	Pendimethalin *	100 ± 0.00	100 ± 0.00	100 ± 0.00	100 ±0.00

*—Standard herbicide, HCCAO—*Hedychium coccineum* aerial part essential oil; HCCRO—*Hedychium coccineum* rhizome part essential oil. Values are means of three replicates ± SD; SD—standard deviation. Within a column, mean values followed by the same letter are not significantly different according to Tukey’s test (*p* < 0.05).

**Table 11:** IC_50_ value of inhibition of root length of HCCAO and HCCRO.

S. No.	Sample Name	IC_50_ Values (µL/mL) in Triplicates			Mean IC_50_ Values (µL/mL) ± SD
	Essential Oil	I	II	III	
1	HCCAO	96.69	96.57	97.29	96.85 ± 0.38
2	HCCRO	93.1	97.85	93.1	94.68 ± 2.74

HCCAO—*Hedychium coccineum* aerial part essential oil; HCCRO—*Hedychium coccineum* rhizome part essential oil; IC_50_—half maximal inhibitory concentration.

**Table 12:** Mean percent inhibition of shoot length of HCCAO and HCCRO.

S. No.	Sample Name	% Inhibition of Shoot Length			
	Essential Oil	50 µL/mL	100 µL/mL	150 µL/mL	200 µL/mL
1.	HCCAO	34.44 ± 1.11 ^b^	52.22 ± 4.00 ^d^	66.66 ± 2.22 ^e^	81.11 ± 2.93 ^g^
2.	HCCRO	40.00 ± 1.11 ^b^	47.77 ± 1.92 ^c^	74.44 ± 1.11 ^f^	99.62 ± 0.64 ^h^
3.	Pendimethalin *	100 ± 0.00	100 ± 0.00	100 ± 0.00	100 ± 0.00

*—Standard herbicide; HCCAO—*Hedychium coccineum* aerial part essential oil; HCCRO—*Hedychium coccineum* rhizome part essential oil; values are means of three replicates ± SD; SD—standard deviation. Within the dataset, mean values with the same letter in superscript are not significantly different, based on Tukey’s test (*p* < 0.05).

**Table 13:** IC_50_ value of inhibition of shoot length of HCCAO and HCCRO.

S. No.	Sample Name	IC_50_ Values (µL/mL) in Triplicates			Mean IC_50_ Values (µL/mL) ± SD
	Essential Oil	I	II	III	
1.	HCCAO	133.76	149.93	115.5	133.06 ± 17.22
2.	HCCRO	86.2	90.85	85.27	87.44 ± 2.98

HCCAO—*Hedychium coccineum* aerial part essential oil; HCCRO—*Hedychium coccineum* rhizome part essential oil IC_50_—half maximal inhibitory concentration.

**Table 14:** Percent mycelial growth inhibition of *F.* *oxysporum*, and *C. lunata* by HCCAO and HCCRO.

Percent Mycelial Growth Inhibition				
Concentration (µL/mL)	*Fusarium* *oxysporum*		*Curvularia lunata*	
	HCCAO	HCCRO	HCCAO	HCCRO
50	15.9 ± 0.64 ^a^	38.1 ± 0.64 ^c^	27.0 ± 0.64 ^b^	18.5 ± 0.67 ^a^
100	32.9 ± 0.64 ^b^	52.6 ± 0.61 ^d^	32.9 ± 0.64 ^c^	32.4 ± 0.29 ^c^
250	54.0 ± 1.69 ^d^	66.7 ± 0.12 ^e^	57.0 ± 0.64 ^d^	42.9 ± 1.69 ^e^
500	69.9 ± 0.57 ^e^	72.7 ± 0.55 ^e^	72.2 ± 1.05 ^f^	58.5 ± 0.57 ^e^
750	83.3 ± 1.11 ^f^	88.1 ±1.28 ^f^	84.1 ± 0.57 ^h^	74.8 ± 0.64 ^g^
Carbendazim *	100 ± 00	100 ± 00	100 ± 00	100 ± 00

*—Standard pesticide; HCCAO—*Hedychium coccineum* aerial part essential oil; HCCRO—*Hedychium coccineum* rhizome part essential oil; SD—Standard deviation. Within the dataset, mean values with same letter in superscript are not significantly different based on Tukey’s test (*p* < 0.05).

**Table 15:** Colony-forming unit (CFL/mL) of *Staphylococcus aureus* and *Salmonella enterica* serovar Typhi by essential oils from the aerial and rhizome part of *H. coccineum*.

Concentration (μL/100 μL)	*Staphylococcus aureus* (log_10_CFU/mL ± SD)		*Salmonella enterica* serovar Typhi (log_10_CFU/mL ± SD)	
	HCCAO	HCCRO	HCCAO	HCCRO
5	1 ± 0 ^g^	1 ± 0 ^h^	6.97 ± 0.41 ^f^	6.34 ± 0.22 ^h^
2.5	2.67 ± 0.11 ^e^	2.079 ± 0.12 ^f^	7.17 ± 0.33 ^e^	6.97 ± 0.37 ^g^
1.25	5.38 ± 0.22 ^d^	5.16 ± 0.34 ^e^	8.00 ± 0.48 ^d^	7.83 ± 0.55 ^e^
0.625	7.38 ± 0.33 ^c^	6.28 ± 0.2 5 ^c^	9.15 ± 0.36 ^c^	9.12 ± 0.39 ^c^
Untreated cells	8.57 ± 0.31 ^a^	8.57 ± 0.31 ^b^	9.16 ± 0.58 ^a^	9.16 ± 0.58 ^b^

HCCAO—*Hedychium coccineum* aerial part essential oil; HCCRO—*Hedychium coccineum* rhizome part essential oil; SD—standard deviation. Within the dataset, mean values with same letter in superscript are not significantly different based on Tukey’s test (*p* < 0.05). CFL—Colony forming unit.

**Table 16:** In silico PASS prediction for antibacterial, antifungal, and nematicidal activity of selected phytochemical compounds from HCCAO and HCCRO.

Pass (Pa > Pi)				
S.No.	Compounds Name	Antibacterial	Antifungal	Nematicidal
1	β-pinene	0.23 > 0.09	0.22 > 0.12	0.24 > 0.16
2	1,8-cineol	0.29 > 0.06	0.24 > 0.12	0.28 > 0.12
3	borneol	0.26 > 0.07	0.34 > 0.06	0.26 > 0.05
4	(*E*)-nerolidol	0.43 > 0.02	0.61 > 0.01	0.36 > 0.02
5	(*E*)-caryophyllene	0.44 > 0.02	0.58 > 0.02	0.48 > 0.01
6	linalool	0.38 > 0.03	0.59 > 0.01	0.37 > 0.02
7	α- pinene	0.32 >0.05	0.43 > 0.04	0.35 > 0.06
8	α-farnesene	0.41 > 0.02	0.60 > 0.01	0.45 > 0.01
9	limonene	0.40 > 0.02	0.58 > 0.02	0.59 > 0.00
10	terpinen-4-ol	0.32 > 0.05	0.46 > 0.03	0.46 > 0.02
11	spathulenol	0.40 > 0.02	0.51 > 0.02	-
12	davanone B	0.45 > 0.02	0.59 > 0.01	0.45 > 0.01
13	γ-eudesmol	0.26 > 0.07	0.28 > 0.08	0.26 > 0.15
14	bulnesol	0.32 > 0.05	0.19 > 0.03	0.21 > 0.19
15	β–eudesmol	0.30 > 0.05	0.40 > 0.04	0.22 > 0.06
16	α-curcumene	0.29 > 0.06	0.44 > 0.04	0.41 > 0.01
17	germacrene D	0.42 > 0.02	0.57 > 0.02	0.45 > 0.00
18	bicyclogermacrene	0.42 > 0.02	0.53 > 0.02	0.63> 0.00
19	7-hydroxyfarnesen	0.44 > 0.02	0.62 > 0.01	0.34 > 0.02
20	β dihydroagarofuran	0.21 > 0.10	0.17 > 0.05	0.32 > 0.09

PASS—prediction of activity spectra for substance; Pa—probable activity; Pi—probable inactivity.

## Data Availability

Not applicable.

## Sample Availability

Sample: *Hedychium coccineum* Buch.-Ham. ex Sm of the compounds is available from the authors.

## References

[1] Fournet J., Sastre C. (2002). Progrès Récents Dans La Connaissance de La Flore de Guadeloupe et de Martinique. Acta Bot. Gall..

[2] Govaerts R. (2009). World Checklist of Selected Plant Families.

[3] Wongsuwan P., Picheansoonthon C. (2011). Taxonomic Revision of the Genus Hedychium J. Koenig (Zingiberaceae) in India. J. R. Inst. Thail..

[4] Jiangyun G., Chunling S., Shuxia Y. (2013). Adaptive Significance of Mass-Flowering in Hedychium Coccineum (Zingiberaceae). Biodivers. Sci..

[5] Quattrocchi U., Quattrocchi U. (2016). CRC World Dictionary of Medicinal and Poisonous Plants.

[6] Tushar S., Basak S., Sarma G.C., Rangan L. (2010). Ethnomedical Uses of Zingiberaceous Plants of Northeast India. J. Ethnopharmacol..

[7] Johnson T., Johnson T. (2019). CRC Ethnobotany Desk Reference.

[8] Basak S., Ramesh A.M., Kesari V., Parida A., Mitra S., Rangan L. (2014). Genetic Diversity and Relationship of Hedychium from Northeast India as Dissected Using PCA Analysis and Hierarchical Clustering. Meta Gene.

[9] Sakhanokho H., Sampson B., Tabanca N., Wedge D., Demirci B., Baser K., Bernier U., Tsikolia M., Agramonte N., Becnel J. (2013). Chemical Composition, Antifungal and Insecticidal Activities of Hedychium Essential Oils. Molecules.

[10] Oliveira M.S., Santana de Oliveira M. (2022). de Essential Oils—Applications and Trends in Food Science and Technology.

[11] Shifah F., Tareq A., Sayeed M., Islam M., Emran T., Ullah M., Mukit M., Ullah A. (2020). Antidiarrheal, Cytotoxic and Thrombolytic Activities of Methanolic Extract of Hedychium Coccineum Leaves. J. Adv. Biotechnol. Exp. Ther..

[12] Nishi S.I., Barua N., Sayeed M.A., Tareq A.M., Mina S.B., Emran T.B., Dhama K. (2021). In vivo and in vitro evaluation of pharmacological activities of hedychium coccineum rhizomes extract. J. Exp. Biol. Agric. Sci..

[13] Rawat A., Prakash O., Kumar R., Arya S., Srivastava R.M. (2021). Hedychium Spicatum Sm.: Chemical Composition with Biological Activities of Methanolic and Ethylacetate Oleoresins from Rhizomes. J. Biol. Act. Prod. from Nat..

[14] Gurib-Fakim A., Maudarbaccus N., Leach D., Doimo L., Wohlmuth H. (2002). Essential Oil Composition of Zingiberaceae Species from Mauritius. J. Essent. Oil Res..

[15] Adams R.P. (2007). Identification of Essential Oil Components by Gas Chromatograpy/Mass Spectrometry, 4th Edition. Illinois USA Allured Publ. Corp. Carol Stream.

[16] Rodrigues T.L.M., Castro G.L.S., Viana R.G., Gurgel E.S.C., Silva S.G., de Oliveira M.S., Andrade E.H. (2020). Physiological Performance and Chemical Compositions of the Eryngium Foetidum L. (Apiaceae) Essential Oil Cultivated with Different Fertilizer Sources. Nat. Prod. Res..

[17] Mesquita K.D.S.M., Feitosa B.D.S., Cruz J.N., Ferreira O.O., Franco C.D.J.P., Cascaes M.M., Oliveira M.S.D., Andrade E.H.D.A. (2021). Chemical Composition and Preliminary Toxicity Evaluation of the Essential Oil from Peperomia Circinnata Link Var. Circinnata. (Piperaceae) in Artemia Salina Leach. Molecules.

[18] Da Silva Júnior O.S., Franco C.D.J.P., de Moraes Â.A.B., Pastore M., Cascaes M.M., do Nascimento L.D., de Oliveira M.S., de Aguiar Andrade E.H. (2022). Chemical Variability of Volatile Concentrate from Two Ipomoea L. Species within a Seasonal Gradient. Nat. Prod. Res..

[19] Silva-Aguayo G., Aguilar-Marcelino L., Cuevas-Padilla E., Loyola-Zapata P., Rodríguez-Maciel J.C., Castañeda-Ramírez G., Figueroa-Cares I. (2021). Essential Oil of Peumus Boldus Molina against the Nematode Haemonchus Contortus (L3) and Three Stored Cereal Insect Pests. Chil. J. Agric. Res..

[20] Kalsi P.S., Bajaj K.L., Mahajan R., Singh P. (1986). Nematicidal Activity of Some Sesquiterpenoids against Rootknot Nematode (Meloidogyne Incognita). Nematologica.

[21] Ntalli N.G., Ferrari F., Giannakou I., Menkissoglu-Spiroudi U. (2010). Phytochemistry and Nematicidal Activity of the Essential Oils from 8 Greek Lamiaceae Aromatic Plants and 13 Terpene Components. J. Agric. Food Chem..

[22] Lahlou M. (2004). Methods to Study the Phytochemistry and Bioactivity of Essential Oils. Phyther. Res..

[23] Amzouar S., Boughdad A., Maatoui A., Allam L. (2016). Comparison of the Chemical Composition and the Insecticidal Activity of Essential Oils of Mentha Pulegium L. Collected from Two Different Regions of Morocco, against Bruchus Rufimanus (Bohman) (Coleoptera: Chrysomelidae). Adv. Environ. Biol..

[24] Kumar R., Kumar R., Prakash O. (2021). Evaluation of In-Vitro Herbicidal Efficacy of Essential Oil and Chloroform Extract of *Limnophila Indica*. Pharma Innov..

[25] Rawat A., Thapa P., Prakash O., Kumar R., Pant A.K., Srivastava R.M., Rawat D.S. (2019). Chemical Composition, Herbicidal, Antifeedant and Cytotoxic Activity of Hedychium Spicatum Sm.: A Zingiberaceous Herb. Trends Phytochem. Res..

[26] Joshi A., Prakash O., Pant A.K., Kumar R., Szczepaniak L., Kucharska-Ambrożej K. (2021). Methyl Eugenol, 1,8-Cineole and Nerolidol Rich Essential Oils with Their Biological Activities from Three Melaleuca Species Growing in Tarai Region of North India. Brazilian Arch. Biol. Technol..

[27] Ray A., Jena S., Dash B., Kar B., Halder T., Chatterjee T., Ghosh B., Panda P.C., Nayak S., Mahapatra N. (2018). Chemical Diversity, Antioxidant and Antimicrobial Activities of the Essential Oils from Indian Populations of Hedychium Coronarium Koen. Ind. Crops Prod..

[28] Spengler G., Gajdács M., Donadu M.G., Usai M., Marchetti M., Ferrari M., Mazzarello V., Zanetti S., Nagy F., Kovács R. (2022). Evaluation of the Antimicrobial and Antivirulent Potential of Essential Oils Isolated from Juniperus Oxycedrus L. Ssp. Macrocarpa Aerial Parts. Microorganisms.

[29] Lin L., Long N., Qiu M., Liu Y., Sun F., Dai M. (2021). The Inhibitory Efficiencies of Geraniol as an Anti-Inflammatory, Antioxidant, and Antibacterial, Natural Agent Against Methicillin-Resistant Staphylococcus Aureus Infection in Vivo. Infect. Drug Resist..

[30] do Vale J.P.C., de Freitas Ribeiro L.H., de Vasconcelos M.A., Sá-Firmino N.C., Pereira A.L., do Nascimento M.F., Rodrigues T.H.S., da Silva P.T., de Sousa K.C., da Silva R.B. (2019). Chemical Composition, Antioxidant, Antimicrobial and Antibiofilm Activities of Vitex Gardneriana Schauer Leaves’s Essential Oil. Microb. Pathog..

[31] Tong S.Y.C., Davis J.S., Eichenberger E., Holland T.L., Fowler V.G. (2015). Staphylococcus Aureus Infections: Epidemiology, Pathophysiology, Clinical Manifestations, and Management. Clin. Microbiol. Rev..

[32] Näsström E., Vu Thieu N.T., Dongol S., Karkey A., Voong Vinh P., Ha Thanh T., Johansson A., Arjyal A., Thwaites G., Dolecek C. (2014). Salmonella Typhi and Salmonella Paratyphi A Elaborate Distinct Systemic Metabolite Signatures during Enteric Fever. Elife.

[33] Mathur R., Oh H., Zhang D., Park S.-G., Seo J., Koblansky A., Hayden M.S., Ghosh S. (2012). A Mouse Model of Salmonella Typhi Infection. Cell.

[34] Mostafa A.A., Al-Askar A.A., Almaary K.S., Dawoud T.M., Sholkamy E.N., Bakri M.M. (2018). Antimicrobial Activity of Some Plant Extracts against Bacterial Strains Causing Food Poisoning Diseases. Saudi J. Biol. Sci..

[35] Epand R.M., Walker C., Epand R.F., Magarvey N.A. (2016). Molecular Mechanisms of Membrane Targeting Antibiotics. Biochim. Biophys. Acta-Biomembr..

[36] Tao N., Liu Y., Zhang M. (2009). Chemical Composition and Antimicrobial Activities of Essential Oil from the Peel of Bingtang Sweet Orange ( Citrus Sinensis Osbeck). Int. J. Food Sci. Technol..

[37] Sabulal B., George V., Dan M., Pradeep N.S. (2007). Chemical Composition and Antimicrobial Activities of the Essential Oils from the Rhizomes of Four Hedychium Species from South India. J. Essent. Oil Res..

[38] Clevenger J.F. (1928). Apparatus for the Determination of Volatile Oil. J. Am. Pharm. Assoc..

[39] Franco C.D.J.P., Ferreira O.O., Antônio Barbosa de Moraes Â., Varela E.L.P., Nascimento L.D.D., Percário S., de Oliveira M.S., Andrade E.H.D.A. (2021). Chemical Composition and Antioxidant Activity of Essential Oils from Eugenia Patrisii Vahl, E. Punicifolia (Kunth) DC., and Myrcia Tomentosa (Aubl.) DC., Leaf of Family Myrtaceae. Molecules.

[40] Ferreira O.O., da Cruz J.N., Franco C.D.J.P., Silva S.G., da Costa W.A., de Oliveira M.S., Andrade E.H.D.A. (2020). First Report on Yield and Chemical Composition of Essential Oil Extracted from Myrcia Eximia DC (Myrtaceae) from the Brazilian Amazon. Molecules.

[41] Eisenback J.D., Sasser J.N., Carter C. (1985). Diagnostic Characters Useful in the Identification of the Four Most Common Species of Root-Knot Nematodes (Meloidogyne Spp.). An Advanced Treatise on Meloidogyne.

[42] Latif R., Abbasi M.W., Zaki M.J., Khan D. (2014). Nemticidal Activity of Bark of Some Tree Species against Root-Knot Nematode Meloidogyne Javanica (Treub) Chitwood. FUUAST J. Biol..

[43] Dawidar A.E.M., Mortada M.M., Raghib H.M., Abdel-Mogib M. (2012). Molluscicidal Activity of Balanites Aegyptiaca against Monacha Cartusiana. Pharm. Biol..

[44] Ferreira Barros A., Paulo Campos V., Lopes de Paula L., Alaís Pedroso L., de Jesus Silva F., Carlos Pereira da Silva J., Ferreira de Oliveira D., Humberto Silva G. (2021). The Role of Cinnamomum Zeylanicum Essential Oil, (E)-Cinnamaldehyde and (E)-Cinnamaldehyde Oxime in the Control of Meloidogyne Incognita. J. Phytopathol..

[45] Tabashnic T., Cushing N.L. (1987). Leaf Residue vs. Topical Bioassays for Assessing Insecticide Resistance in the Diamond-Back Moth, *Plutella Xylostella* L.. FAO Plant Prot. Bull..

[46] Eloff J. (1998). Which Extractant Should Be Used for the Screening and Isolation of Antimicrobial Components from Plants?. J. Ethnopharmacol..

[47] Sahu A., Devkota A. (2013). Allelopathic Effects of Aqueous Extract of Leaves of Mikania Micrantha H.B.K. on Seed Germination and Seedling Growht of *Oryza Sativa* L. and *Raphanus Sativus* L.. Sci. World.

[48] Batista C.D.C.R., de Oliveira M.S., Araújo M.E., Rodrigues A.M., Botelho J.R.S., da Silva Souza Filho A.P., Machado N.T., Junior R.N.C. (2015). Supercritical CO2 Extraction of Açaí (Euterpe Oleracea) Berry Oil: Global Yield, Fatty Acids, Allelopathic Activities, and Determination of Phenolic and Anthocyanins Total Compounds in the Residual Pulp. J. Supercrit. Fluids.

[49] de Oliveira M.S., da Costa W.A., Pereira D.S., Botelho J.R.S., de Alencar Menezes T.O., de Aguiar Andrade E.H., da Silva S.H.M., da Silva Sousa Filho A.P., de Carvalho R.N. (2016). Chemical Composition and Phytotoxic Activity of Clove (Syzygium Aromaticum) Essential Oil Obtained with Supercritical CO_2_. J. Supercrit. Fluids.

[50] Gurgel E.S.C., de Oliveira M.S., Souza M.C., da Silva S.G., de Mendonça M.S., da Silva Souza Filho A.P. (2019). Chemical Compositions and Herbicidal (Phytotoxic) Activity of Essential Oils of Three Copaifera Species (Leguminosae-Caesalpinoideae) from Amazon-Brazil. Ind. Crops Prod..

[51] Goswami S., Kanyal J., Prakash O., Kumar R., Rawat D.S., Srivastava R.M., Pant A.K. (2019). Chemical Composition, Antioxidant, Antifungal and Antifeedant Activity of the Salvia Reflexa Hornem. Essential Oil. Asian J. Appl. Sci..

[52] (CLSI) Clinical and Laboratory Standards Institute (2006). Methods for Dilution Antimicrobial Susceptibility Tests for Bacteria That Grow Aerobically.

[53] Ferreira O.O., da Silva S.H.M., de Oliveira M.S., de Andrade E.H.A. (2021). Chemical Composition and Antifungal Activity of Myrcia Multiflora and Eugenia Florida Essential Oils. Molecules.

[54] Mesomo M.C., Corazza M.L., Ndiaye P.M., Dalla Santa O.R., Cardozo L., Scheer A.D.P. (2013). Supercritical CO_2_ extracts and Essential Oil of Ginger (Zingiber Officinale R.): Chemical Composition and Antibacterial Activity. J. Supercrit. Fluids.

[55] Filimonov D.A., Lagunin A.A., Gloriozova T.A., Rudik A.V., Druzhilovskii D.S., Pogodin P.V., Poroikov V.V. (2014). Prediction of the Biological Activity Spectra of Organic Compounds Using the Pass Online Web Resource. Chem. Heterocycl. Compd..

[56] Concato J., Hartigan J.A. (2016). P Values: From Suggestion to Superstition. J. Investig. Med..

